# Avens Root (*Geum Urbanum* L.) Extract Discovered by Target-Based Screening Exhibits Antidiabetic Activity in the Hen’s Egg Test Model and *Drosophila melanogaster*

**DOI:** 10.3389/fphar.2021.794404

**Published:** 2021-12-15

**Authors:** Ilka Günther, Gerald Rimbach, Sandra Nevermann, Cathrina Neuhauser, Verena Stadlbauer, Bettina Schwarzinger, Clemens Schwarzinger, Ignacio R. Ipharraguerre, Julian Weghuber, Kai Lüersen

**Affiliations:** ^1^ Institute of Human Nutrition and Food Science, University of Kiel, Kiel, Germany; ^2^ School of Engineering, University of Applied Sciences Upper Austria, Wels, Austria; ^3^ FFoQSI - Austrian Competence Centre for Feed and Food Quality, Safety and Innovation, Tulln, Austria; ^4^ Institute for Chemical Technology of Organic Materials, Johannes Kepler University, Linz, Austria

**Keywords:** avens root, antidiabetic, hen’s egg test, *Drosophila melanogaster*, α-glucosidase, glucose transporter 4, ussing chamber, sodium-dependent glucose transporter 1

## Abstract

Medicinal plant extracts are becoming increasingly important as an alternative for traditional drugs against diabetes mellitus (DM). For this reason, we initialized a target-based screening of 111 root extracts from an open access plant extract library (PECKISH) by ascertaining their *in-vitro* inhibitory efficacy on α-glucosidase. The two most active extracts *Geum urbanum* L. (roseroot) and *Rhodiola rosea* L. (avens root) were further tested for their antidiabetic activities in terms of their impact on different regulatory key points of glucose homeostasis. To this end, various enzyme- and cell culture-based *in-vitro* assays were employed including the determination of sodium-dependent glucose transporter 1 (SGLT1) activity in Caco-2 monolayers by Ussing chambers and of glucose transporter 4 (GLUT4) translocation in a GFP-reporter cell line. Subsequently, the antidiabetic potential of the root extracts were further evaluated in *in-vivo* models, namely hen’s eggs test and the fruit fly *Drosophila melanogaster*. Avens root extract was found to be a more potent inhibitor of the enzymes α-glucosidase and dipeptidyl peptidase-4 (DPP4) than roseroot extract. Most importantly, only avens root extract exhibited antidiabetic activity in the two *in-vivo* models eliciting a reduced blood glucose level in the *in-ovo* model and a decline of the triglyceride level in a dietary starch-induced *D. melanogaster* obesity model. Analyses of the polyphenolic composition of the avens root extract by HPLC revealed a high content of ellagic acid and its derivatives as well as ellagitannins such as pedunculagin, stenophyllanin, stachyurin, casuarinin and gemin A. In conclusion, avens root extract represents a promising medicinal plant that should be considered in further *in-vivo* studies on hyperglycemia in laboratory rodents and humans.

## Introduction

Diabetes mellitus (DM) and its consequences have become an increasing health problem worldwide and a considerable financial burden. In 2019, the total health expenditures for diabetes were estimated to be approximately USD 760 billion (International Diabetes Federation, 2019). Hyperglycemia is the hallmark of DM and commonly triggers diabetic complications such as damage or dysfunction of various organs (Agwaya et al., 2016). Accordingly, the reduction of postprandial blood glucose elevation is a relevant therapeutic strategy for the prevention and control of DM. An additional problem is that the drugs currently used for DM treatment are not only expensive but also cause side effects (American Diabetes Association, 2015; Padhi et al., 2020). Natural resources, especially medicinal plants have been used traditionally for DM treatment and may be considered as alternatives to the drugs available on the market (Arulselvan et al., 2014; Eddouks et al., 2014; Salehi et al., 2019). In fact, numerous studies have demonstrated antihyperglycemic effects for certain medicinal plant extracts, however, often neglected to elucidate the underlying cellular and molecular mechanisms in more detail (Eddouks et al., 2014). Modulation of the blood glucose level can be achieved by targeting various hormones, enzymes and transporters along the carbohydrate digestion/absorption pathway including intestinal α-amylase, α-glucosidase, sodium-dependent glucose transporter 1 (SGLT1) as well as the insulin secretion machinery or glucose transporter 4 (GLUT4) in peripheral tissues (Padhi et al., 2020). In this regard, studies, which systematically investigate whether plant extracts act on more than one of these targets, are of interest. Consequently, more studies are needed to 1) evaluate known traditional medicinal plants, 2) identify novel plant material with antidiabetic properties, 3) elucidate the underlying molecular mechanisms of action and 4) characterize the biologically active compounds.

In target-based screening approaches, plant extracts are usually tested by *in-vitro* enzyme and/or cell culture-based assays. Although these methods allow high-throughput screening and reveal a first notion on the putative bioactivity, the informative value must be handled with caution, since one has to be aware that crucial aspects of pharmacology such as bioavailability, biotransformation and excretion are not covered (Croston, 2017). Hence, *in-vivo* models are required to validate the effects found in *in-vitro* experiments. However, studies with traditional model organisms, in particular rodents, are expensive, laborious, time-consuming and raise ethical concerns. Hence, alternative models such as the recently introduced modified hen’s egg test (Gluc-HET), which enables testing of bioactives and plant extracts on insulin-mimetic properties *in-ovo*, have been developed (Haselgrübler et al., 2018b). Moreover, we propose that the use of invertebrate models such as the fruit fly *Drosophila melanogaster* is another option to precede/replace rodent studies. *D. melanogaster* has become a versatile model in nutritional research (Staats et al., 2018), especially, since central metabolic and regulatory pathways including metabolism of carbohydrates, lipids, and insulin signaling are evolutionary conserved (Chatterjee and Perrimon, 2021). Accordingly, fruit flies have been successfully used to elucidate the impact of plant bioactives on the energy metabolism and to identify potential molecular targets such as α-amylase and α-glucosidase (Wagner et al., 2015). Moreover, a high sugar diet was found to be sufficient to induce an obesity and insulin-resistance phenotype in the fruit fly (Musselman et al., 2011). The composition of *Drosophila* diets can be adapted to specific research objectives, since recipes for different complex and chemically defined diets are available (Lüersen et al., 2019). In this regard, it is of note that varying the content of a starch-based diet has been recently reported to affect the triglyceride level of fruit flies. Most important, high starch level elicited an obese phenotype (Abrat et al., 2018). Hence, when employing this *Drosophila* starch-based medium, we assume that numerous of the abovementioned aspects of carbohydrate digestion/absorption are addressed in plant extract supplementation studies with the final read out lipid storage determination.

In general, studies on potential medicinal plants starts with the selection of the appropriate plant material and extraction method (Azwanida, 2015; Abubakar and Haque, 2020). Plant extract collections represent an enormous help by enabling the research community to screen a large amount of plant extracts with specific properties. The plant extract collection Kiel in Schleswig-Holstein (PECKISH) is an open access screening library, containing extracts from over 880 different plant species and 11 different plant tissues (Onur et al., 2013).

In this study, we initially carried out a target-based screening of 111 extracts from root material derived from the PECKISH library for *in-vitro* α-glucosidase inhibition to discover and analyze extracts with promising antidiabetic effects. The two most potent α-glucosidase inhibitors, aqueous extracts from *Geum urbanum* L. (avens root) and *Rhodiola rosea* L. (roseroot) were further examined by *in-vitro* enzyme and cell culture-based assays as well as by *in-ovo* (hen’s egg test-chorioallantoic membrane; HET-CAM) and *in-vivo* models (*D. melanogaster*) to pinpoint potential antidiabetic activities. Accordingly, starch digestion by α-amylase (Ozougwu and Barnabas, 2018), intestinal glucose transport by sodium-dependent glucose transporter 1 (SGLT1) (Song et al., 2016), the incretin system in terms of dipeptidyl peptidase-4 (DPP4) inhibition (Papaetis, 2014), the glucose transport in peripheral tissues by glucose transporter 4 (GLUT4) translocation (Govers, 2014), insulin mimetic effects (Haselgrübler et al., 2017; 2018b), and the impact on dietary carbohydrate-related lipid storage were investigated. High-performance liquid chromatography-mass spectrometry (HPLC-MS) analysis of the extracts revealed main polyphenolic constituents with putative biological activity.

## Materials and Methods

### Plant Extract Screening Library and Root Extract Preparation

For the initial screening, we selected 111 ethanolic and aqueous extracts of the PECKISH library that derive from plant root material (Onur et al., 2013). For further studies, the aqueous root extracts of *G. urbanum* (avens root, Kräuterhaus, Hamburg, Germany) and *R. rosea* (roseroot, Kräuter-Pflug, Kiel, Germany) were freshly prepared according to the protocol described in Onur et al., 2013. In brief, the dried raw plant material was grinded by IKA analytical mill Type A11 basic (IKA-Werke, Staufen, Germany). Three gram of the grinded material and 30 ml of boiling double distilled water (Rotipuran ≥99.8% p.a.; Carl Roth, Karlsruhe, Germany) were stirred gently for 1 min, followed by sonication for 1 min (Sonoplus UW 2070; Bandelin electronic, Berlin, Germany). Tubes were centrifuged (Centrifuge 5,702; Eppendorf, Hamburg, Germany) for 2 min at 2,000 × g, before the supernatant was filtered by a folded filter (MN 615¼, 185 mm; Macherey-Nagel, Düren, Germany). The aqueous extracts were stored at −20°C.

### *In-Vitro* α-Glucosidase Inhibition Assay

The spectrophotometric assay was conducted as previously described (Awosika and Aluko, 2019) with some modifications. Root extracts were diluted with ultrapure water (Purelab Flex, ELGA Veolia, United Kingdom) to give concentrations of 10 µg/ml to 1,000 μg/ml. Of these dilutions, 15 µl were mixed in 96-well microtest plates (VWR, Darmstadt, Germany) with 105 µl of 0.1 M phosphate buffer, pH 6.8 and 15 µl of α-glucosidase (0.5 U/ml) from *Saccharomyces cerevisiae* (Sigma-Aldrich, Taufkirchen, Germany). Following 5 min pre-incubation at 37°C, 15 µl 10 mM p-nitrophenyl-α-D-glucopyranoside (Sigma-Aldrich, Taufkirchen, Germany) in the same buffer were added as a substrate to initiate the reaction. The mixture was incubated for 20 min at 37°C, before 50 µl 2 M Na_2_CO_3_ (VWR, Darmstadt, Germany) were added to stop the reaction. A microplate reader (iEMS Reader MF, MTX Lab Systems, Helsinki, Finland) was used to measure the absorbance at 405 nm. Acarbose (Sigma-Aldrich, Taufkirchen, Germany) served as a reference inhibitor. The percentage inhibition of α-glucosidase was calculated by using the following equation:

$$Inhibition\left(\%\right)=\;\frac{\left[\left(AbC-AbC_{blank}\right)-\left(AbS-AbS_{blank}\right)\right]}{\left(AbC-AbC_{blank}\right)}\times 100$$

AbC, absorbance of the control; AbS, absorbance of the sample. IC_50_ values were calculated by nonlinear regression using GraphPad Prism version 8.1.1.

### *In-Vitro* α-Amylase Inhibition Assays

#### α-Amylase Disc Assay

The α-amylase disc assay was conducted according to (Correia et al., 2004) with minor modifications. Root extracts were diluted 1:4, 1:16 and 1:64 with ultrapure water (Purelab Flex, ELGA Veolia, United Kingdom). 80 µl of these dilutions or, as control, ultrapure water was mixed with 20 µl porcine pancreatic α-amylase (Sigma-Aldrich, Taufkirchen, Germany) (20 mg/ml 20 mM sodium phosphate buffer, pH 6.9). Acarbose added at concentrations of 0.1, 1, and 10 mM was used as a reference inhibitor. Four filter discs (diameter of 0.5 cm) were placed on Petri plates (92 × 16 mm, Sarstedt, Nürnbrecht, Germany) filled with medium containing 1% agar-agar (Carl Roth, Karlsruhe, Germany) and 1% starch (VWR, Darmstadt, Germany). 20 µl of the mixtures were given onto a filter disc and the plates were incubated overnight at 37°C. After removing the filter discs, 5 mL of iodine stain solution (5 mM iodine in 3% potassium iodide solution, both chemicals from Merck, Darmstadt, Germany) were added to each plate. Following 15 min incubation excess iodine stain was drained, before the diameter of the cleared zones was measured. The percentage inhibition of α-amylase at each extract concentration was calculated by using the following equation:

$$Inhibition\left(\%\right)=\left(1-\frac{Diameter\;of\;sample}{Diameter\;of\;control}\right)\times 100$$

#### α-Amylase Spectrophotometric Assay

The spectrophotometric assay was conducted according to the method described by Apostolidis et al. (2006) with minor modifications. Root extracts were diluted with ultrapure water (Purelab Fles, ELGA Veolina, United Kingdom) to give concentrations of 10–20,000 μg/ml. Of these dilutions, 50 µl were mixed with 50 µl of α-amylase in 20 mM sodium phosphate buffer, pH 6.9 (0.5 mg/ml). Following 10 min pre-incubation at 25°C, 50 µl 1% starch solution that had been cooked for 15 min in the same buffer were added. The mixture was incubated for 10 min at 25°C. Thereafter, 100 µl of a color reagent (1% 3,5-dinitrosalicylic acid and 30% sodium potassium tartrate in 0.4 M NaOH, all chemicals from Sigma-Aldrich, Taufkirchen, Germany) were added. The mixture was incubated for an additional 5 min at 100°C and cooled to room temperature, before the absorbance was measured at 540 nm by a microplate reader (iEMS Reader MF, MTX Lab Systems, Helsinki, Finland). Acarbose was used as a reference inhibitor. The percentage inhibition of α-amylase was calculated by using the following equation:

$$Inhibition\left(\%\right)=\;\frac{\left[\left(AbC-AbC_{blank}\right)-\left(AbS-AbS_{blank}\right)\right]}{\left(AbC-AbC_{blank}\right)}\times 100$$

AbC, absorbance of the control; AbS, absorbance of the sample. IC_50_ values were calculated by nonlinear regression using GraphPad Prism version 8.1.1.

### Cell Culture

The Caco-2/PD7 clone was kindly provided by Edith Brot-Laroche (Unité de Recherches sur la Différenciation Cellulaire Intestinale, Villejuif Cedex, France) (Mahraoui et al., 1994). Caco-2/PD7 cells were maintained in high-glucose DMEM (PAN Biotech GmbH) supplemented with 20% (v/v) fetal bovine serum (FBS,Thermo Fisher Scientific, life technologies™, Darmstadt, Germany) and 1% penicillin/streptomycin (PAN Biotech, Aidenbach, Germany).

HeLa-GLUT4-myc-GFP cells were maintained in RPMI 1640 medium (2,000 μg/ml NaHCO_3_, stable glutamine, low endotoxin) supplemented with 10% FBS, 1% penicillin/streptomycin and 1% G418 (PAN-Biotech, Aidenbach, Germany). All cells were grown at 37°C in a humidified atmosphere with 5% CO_2_.

### Ussing Chamber Experiments

Caco-2/PD7 cells were seeded into 6-well Corning^®^ Costar^®^ Snapwell cell culture inserts (0.4 μm pore size, 1.12 cm^2^ surface area) (Merck, Darmstadt, Germany) at a density of 1 × 10^6^ cells/well. 0.5 ml of the cell-containing medium were given into the upper compartment (apical side) and 2.5 ml of cell-free medium into the lower compartment (basolateral side). Cells were cultured in plates for 21 days and medium was replaced every other day. After 7 days the apical medium was modified, now lacking FBS in order to mimic the physiological situation and to support the process of polarization (Ferruzza et al., 2012).

Transepithelial electrical resistance (TEER) of the Caco-2/PD7 monolayer was measured against a blank well containing cell culture medium only, using a Millicell ERS-2 Volt-Ohm Meter equipped with a STX01 planar electrode (Merck, Darmstadt, Germany). Only monolayers with TEER values exceeding 400 Ω cm^2^ were considered as functional barriers and were used in transport studies (Stockdale et al., 2019).

SGLT1 inhibitory activity of extracts were examined by employing Ussing chambers (EasyMount Diffusion Chamber System, Physiologic Instruments, San Diego, CA, United States) following the protocols described in (Clarke, 2009; Schloesser et al., 2017) with modifications. Images of the experimental procedure can be found in Supplementary Figure S1. Prior to the experiments, half-chambers were filled with 5 mL of Hank’s balanced salt solution (HBSS) containing 140 mmol/L NaCl, 5 mmol/L KCl, 1 mmol/L CaCl_2_, 0.4 mmol/L MgSO_4_, 0.5 mmol/L MgCl_2_, 0.3 mmol/L Na_2_HPO_4_, 0.4 mmol/L KH_2_PO_4_, 4 mmol/L NaHCO_3_ (pH 7.2). The HBSS in the chambers was heated to 37°C and oxygenated by influx of carbogen-gas (95% oxygen, 5% carbon dioxide).

Caco-2/PD7 monolayers were washed from both sides with 37°C warm HBSS before mounting the Snapwell inserts in Ussing chamber slides (P2302). Both half-chambers were refilled with 5 ml HBSS solution containing 10 mmol/L mannitol apically and 10 mmol/L glucose basolaterally, maintained at 37°C and continuously carbogen bubbled. The transepithelial potential difference was continuously monitored under open circuit conditions using a DVC 1000 amplifier (WPI) and recorded through Ag-AgCl electrodes and HBSS agarose bridges (4%). Subsequently, the short-circuit current (I_SC_; μA cm^−2^) was measured via an automatic voltage clamp (VCC MC8 MultiChannel Voltage-Current Clamp; Physiologic Instruments, San Diego, CA, United States). Recordings were collected and stored using the A&A II (Acquire & Analyze Data) acquisition software (Physiological Instruments, San Diego, CA, United States).

Transepithelial resistance and I_SC_ were allowed to stabilize for approximately 10–20 min. After that, 10 mmol/L glucose was added apically to stimulate Na^+^-coupled glucose transport and, for osmotic reasons, 10 mmol/L mannitol was given simultaneously to the basolateral side. When the glucose-stimulated I_SC_ reached a stable plateau (usually within 10 min), root extracts at a final concentration of 1,000 μg/ml or phlorizin at a final concentration of 0.1 mM as positive control was added to the apical and basolateral side of the chambers. I_SC_ values were further recorded until they reached a stable level (again usually within 10 min). The average I_SC_ of 2 min intervals within stable plateaus were used to calculate differences in SGLT1 transport activity. The ΔI_SC_(1) value that indicates intestinal SGLT1-dependent glucose transport was calculated by the difference: I_SC_ (after apical addition of glucose) - I_SC_ (before apical addition of glucose). ΔI_SC_(2) values indicating SGLT1 inhibition were calculated by the difference: I_SC_ (after the addition of the inhibitor) – I_SC_ (prior to apical addition of glucose). Finally, the inhibitory activity was calculated by using the following equation:

$$Inhibition\left(\%\right)=\left(1-\frac{\Delta ISC\left(2\right)}{\Delta ISC\left(1\right)}\right)\times 100$$

### DPP4 Inhibition Assay

The DPP4 inhibitor activity of selected root extracts tested at a final concentration of 1,000 μg/ml was determined with the DPP4 inhibitor screening kit according to manufacturer instructions (Sigma-Aldrich, Taufkirchen, Germany). In brief, 50 µl of inhibition reaction mix, containing 49 µl assay buffer and 1 µl DPP4 enzyme, were mixed in black 96-well microtiter plates with 25 µl of root extracts or the reference inhibitor sitagliptin. Following 10 min pre-incubation at 37°C, 25 µl of an enzymatic reaction mix containing 23 µl assay buffer and 2 µl substrate was added to each well. The fluorescence signal (excitation wavelength of 360 nm, emission wavelength of 465 nm) was measured at 37°C over 30 min in 1 min intervals.

### Total Internal Reflection Fluorescence Microscopy

TIRF microscopy was used to determine the effect of root extracts on GLUT4 translocation (Lanzerstorfer et al., 2014; Stadlbauer et al., 2016, 2020). HeLa-GLUT4-myc-GFP cells were seeded into 96-well imaging plates (40,000 cells/well) and grown overnight, followed by removal of growth medium, washing with HBSS buffer (PAN-Biotech, Aidenbach, Germany) and starvation for 3 h in HBSS buffer. The cells were incubated with insulin (100 nM; Sigma Aldrich, Schnelldorf, Germany) or selected root extracts (1:10,000) dissolved in Krebs Ringer phosphate HEPES buffer (KRPH; 20 mM HEPES, 1 mM CaCl_2_, 136 mM NaCl, 4.7 mM KCl, 1 mM MgSO_4_ and 5 mM KH_2_PO_4_ at pH 7.4). Images were taken using the automated TIRF function of Nikon Eclipse Ti2 microscope at time-points 20, 10, and 0 min before and 10, 20, and 30 min after the addition of insulin, KRPH or root extracts to monitor GFP signal changes. 25 images per well were taken over a total time-range of 50 min. Analysis of images was done using the intensity analysis tool from SPOTTY (Spotty, 2021). Results were derived from subtraction of image background signal from raw data. Values were averaged and the percentage of signal change was calculated. Graphs were generated by using GraphPad Prism.

### Hen’s Egg Test-Chorioallantoic Membrane

The HET-CAM test was used as previously reported (Haselgrübler et al., 2017; 2018b; 2018a). Images of the experimental procedure, reproduced from Stadlbauer et al., 2021, can be found in (Supplementary Figure S2). Furthermore, a video of the experiment is available in Haselgrübler et al., 2018b. Briefly, eggs were incubated at 38°C for 11°days. The eggs were automatically and constantly turned, checked for fertilization via candling, and the air bladder area was marked. The eggshell was lightly pecked with a pointed pair of tweezers in this area and 300 µl of a solution containing the putative blood glucose-lowering substance were applied with a syringe into the air compartment of the egg. Root extracts were given at a final concentration of 1:17. 3°U/ml Novo Rapid (Novo Nordisk, Bagsvaerd, Denmark) was used as positive and dH_2_O as negative control. The eggs were placed back in the incubator for 60 min. After incubation, the eggshell above the air bladder was carefully removed and the eggshell membrane was equilibrated with PBS (PAN-Biotech, Aidenbach, Germany). In the next step, the eggshell membrane was removed and the chorioallantoic membrane was carefully cut with a micro-scissor. A suitable blood vessel was cut, and leaking blood was collected. The blood glucose levels were determined via a blood glucose meter (Accu-Chek Performa, Roche Diabetes Care GmbH, Mannheim, Germany). For each time point, at least 10 fertilized eggs were used. Each experiment was repeated at least 2–4 times.

### Dietary Starch-Induced *Drosophila melanogaster* Obesity Model

The *D. melanogaster* wild type strain *w^1118^* [(Bloomington *Drosophila* Stock Center #5905, Indiana University, Bloomington, United States] was used in feeding studies. Fruit flies were maintained on Caltech medium consisting of 5.5% dextrose, 3.0% sucrose, 6.0% cornmeal, 2.5% inactive dry yeast, 1.0% agar Type II (Kisker, Steinfurt, Germany) with 0.15% Tegosept (Genesee Scientific, San Diego, United States), and 0.3% propionic acid (Carl Roth, Karlsruhe, Germany) serving as preservatives. The animals were cultured in climate cabinets (HPP750 or HPP110, Memmert, Schwabach, Germany) under standard conditions at 25°C of ambient temperature, 60% humidity, and a 12/12 h light/dark cycle as previously described (Staats et al., 2019; Kaufman et al., 2021). Synchronized eggs were given onto a starch-based control diet 10% soluble starch (VWR, Darmstadt, Germany), 4% yeast, 1% agar, 0.18% nipagin according to Abrat et al., 2018 or experimental diets that were additionally supplemented with 2.5% of the selected root extracts or 1.8 μg/ml acarbose. After larval development, pupation and eclosion, flies were synchronized and mated for 2°days. On day 3 after eclosion, mated female flies were sorted and further maintained by transferring the flies to the respective fresh experimental media every other day, before they were harvested on day 10. After determining their wet weights, 10 flies were homogenized in PBS containing 0.05% Triton X100 for 10 min at 4°C and 25 Hz using a tissue lyser (Qiagen TissueLyser II, Hilden, Germany). The triglyceride and protein content of the fly lysates were determined by employing Infinity triglycerides reagent (Thermo Fisher Scientific, Waltham, United States) and the Pierce BCA Protein Assay Kit (Pierce Biotechnology, Rockford, United States), respectively.

### Determination of Total Phenolic Content

Total phenolic contents (TPC) of the root extracts were determined by the Folin-Ciocalteu method according to (Shetty et al., 1995; Kwon et al., 2006). In brief, 100 µl extract solution (1,250 μg/ml) were mixed with 100 µl of 95% ethanol, 500 µl of distilled water and 50 µl of diluted Folin-Ciocalteu reagent (1:2, v/v, Merck, Darmstadt, Germany). The mixture was incubated for 5 min before 100 µl of 5% Na_2_CO_3_ were added. The reaction mixture was incubated in the dark for 45 min and the absorbance was determined at 725 nm. TPC of the extracts were expressed in mg of gallic acid equivalents (GAE) per L using a calibration curve of gallic acid (Sigma-Aldrich, Taufkirchen, Germany).

### HPLC-MS Analysis

High-resolution mass spectra were obtained using a Thermo Scientific LTQ Orbitrap Velios with an electrospray as well as an APCI source operated in positive and negative ionization mode. Separations were performed using a Thermo Scientific Surveyor HPLC equipped with an Accucore C18 column (150 mm × 3.0°mm, 2.6 μm particle size; Thermo Scientific) The column temperature was set to 40°C and the injection volume was 1 µl. Preconnected to MS analyses, absorbance was monitored at 260 nm by using an FLD-34000RS diode array detector (DAD). The analytes were separated by gradient elution with mobile phase A containing 0.1% formic acid (FA) in water and mobile phase B containing 0.1% FA in acetonitrile at a flow rate of 0.5 ml/min. The elution gradient starting conditions were 95% A and 80% B at 17 min for 3 min. B was reduced to 5% at 20 min until 25 min. The resolution was set to 30,000 and diisooctylphthalate (m/z = 391.2843) was used as internal standard for mass calibration. Spectra were collected from 100–1,000°m/z and MS2 spectra were automatically recorded from the most intense peaks. Identification based on high-resolution MS data and comparison to literature (Granica et al., 2016; Olennikov et al., 2020).

### Statistics

Ussing chamber experiments were analyzed with a two-sided paired Students t-test. *p*-values less than 0.05 were considered significant. Statistical analyses of the DPP4 inhibition assay was performed using the software R version 3.4.3 (R-Core-Team, 2015) and an appropriate mixed model (Laird and Ware, 1982; Verbeke and Molenberghs, 2000). Normal distribution was determined with the Shapiro-Wilk Test and an analysis of variances (ANOVA) followed by a post-hoc multiple comparison test of Dunnett (Bretz et al., 2010). Statistical analysis for GLUT4 microscopy and *in-ovo* experiments was performed using an unpaired *t*-test in GraphPad Prism (version 6.02, GraphPad Software Inc., San Diego, CA, United States). *D. melanogaster* experiments were analyzed by using one-way ANOVA followed by Dunnett’s multiple comparisons test in GraphPad Prism.

## Results

### Avens Root Extract, a Potent Inhibitor of α-Glucosidase But Not of α-Amylase

Of the 111 root samples of the local PECKISH extract library that were screened for α-glucosidase inhibition, the aqueous extracts of *G. urbanum* (avens root) and *R. rosea* (roseroot) were found to be most potent and, hence, were selected for further studies. α-Glucosidase activity was inhibited by both extracts in a dose-dependent manner resulting in IC_50_ values of 3.76 μg/ml for avens root and 5.51 μg/ml for roseroot. Accordingly, the root extracts are approximately 90 (roseroot) and 130 (avens root) times more potent in inhibiting the α-glucosidase activity than the positive control acarbose with an IC_50_ value of 493 μg/ml (Table 1). When tested in combination, the root extracts *G. urbanum* and *R. rosea* exhibited additive but not synergistic activity with respect to α-glucosidase inhibition (Table 2).

**TABLE 1:** IC_50_ values for the *in-vitro* inhibition of α-amylase and α-glucosidase by root extracts.

Sample	α-amylase	α-glucosidase
	IC_50_ [µg/ml] range	IC_50_ [µg/ml] range
Acarbose	15.7	493
	12.7–18.6	348–697
Avens root	>300	3.76
		2.58–4.78
Roseroot	13.6	5.51
	9.2–17.4	3.38–7.70

Enzyme activity was determined spectrophotometrically. Values include data of three independent experiments and were calculated by using GraphPad Prism.

**TABLE 2:** Combined effects of root extracts on *in-vitro* activity of α-glucosidase.

Sample	α-glucosidase inhibition [%]
3.76 μg/ml avens root	79.4 ± 6.4
5.51 μg/ml roseroot	79.8 ± 6.6
1.88 μg/ml avens root	33.3 ± 4.0
2.76 μg/ml roseroot	44.8 ± 8.1
1.88 μg/ml avens root + 2.76 μg/ml roseroot	74.7 ± 8.5

Avens root and roseroot extracts were tested for their effects on α-glucosidase activity at concentrations corresponding to the calculated IC_50_ values given in Table 1 (3.76 and 5.51 μg/ml) and the respective half values. To elucidate putative synergistic effects, both extracts were also applied in combination at concentrations representing their calculated half IC_50_ values. Data represent means ± SEM of three independent determinations.

**FIGURE 1:**
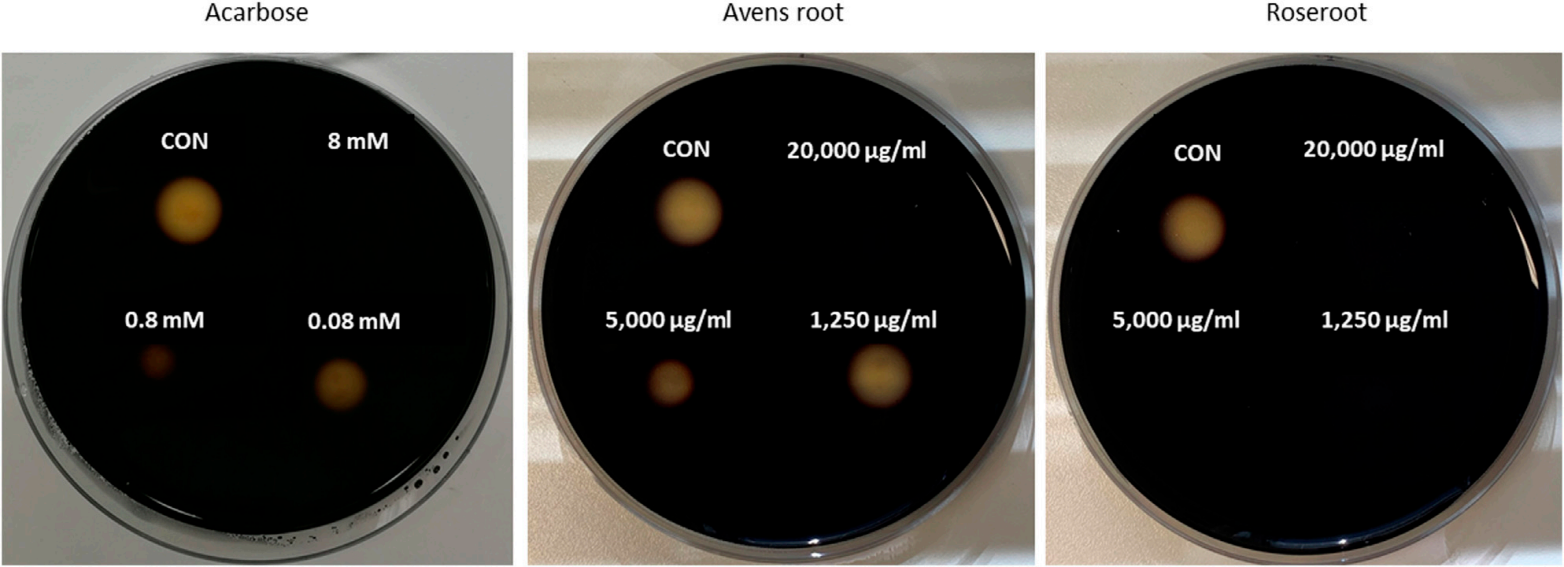
Inhibition of porcine α–amylase *in-vitro* activity by root extracts. Filter discs (diameter of 0.5 cm) were placed on Petri plates that were filled with medium containing 1% agar-agar and 1% starch. Porcine pancreatic α-amylase was first mixed with decreasing concentrations of the reference inhibitor acarbose, avens root or roseroot extract (final concentrations are indicated) and then given onto the filter discs. The plates were incubated overnight at 37°C, before the filter discs were removed. Following iodide-staining, the inhibition of α-amylase was calculated by comparing the diameter of the cleared zones of control filter discs (CON, α-amylase alone) with those of filter discs where the tested root extract or acarbose was added to α-amylase. The smaller the diameter of the cleared zone was, the stronger was the inhibition of the α-amylase activity. Exemplary results are shown.

Remarkably, marked differences were observed in α-amylase-inhibitory activities between the two root extracts (Figure 1; Table 1). Roseroot extract at a final concentration of 300 μg/ml led to an efficient inhibition of α-amylase activity by 90.4%, which is comparable to the effect of the reference inhibitor acarbose (89.0% inhibition at the same concentration). In good accordance, the IC_50_ value for the roseroot extract was calculated to be 13.6 μg/ml, which is similar to the value obtained for acarbose (15.7 μg/ml) (Table 1). In contrast to that, avens root did not influence α-amylase activity up to a final concentration of 300 μg/ml and noticeable inhibition was achieved solely when concentration beyond 1,250 μg/ml were applied (Figure 1).

### Avens Root Extract, an Inhibitor of SGLT1-Mediated Glucose Transport

**FIGURE 2:**
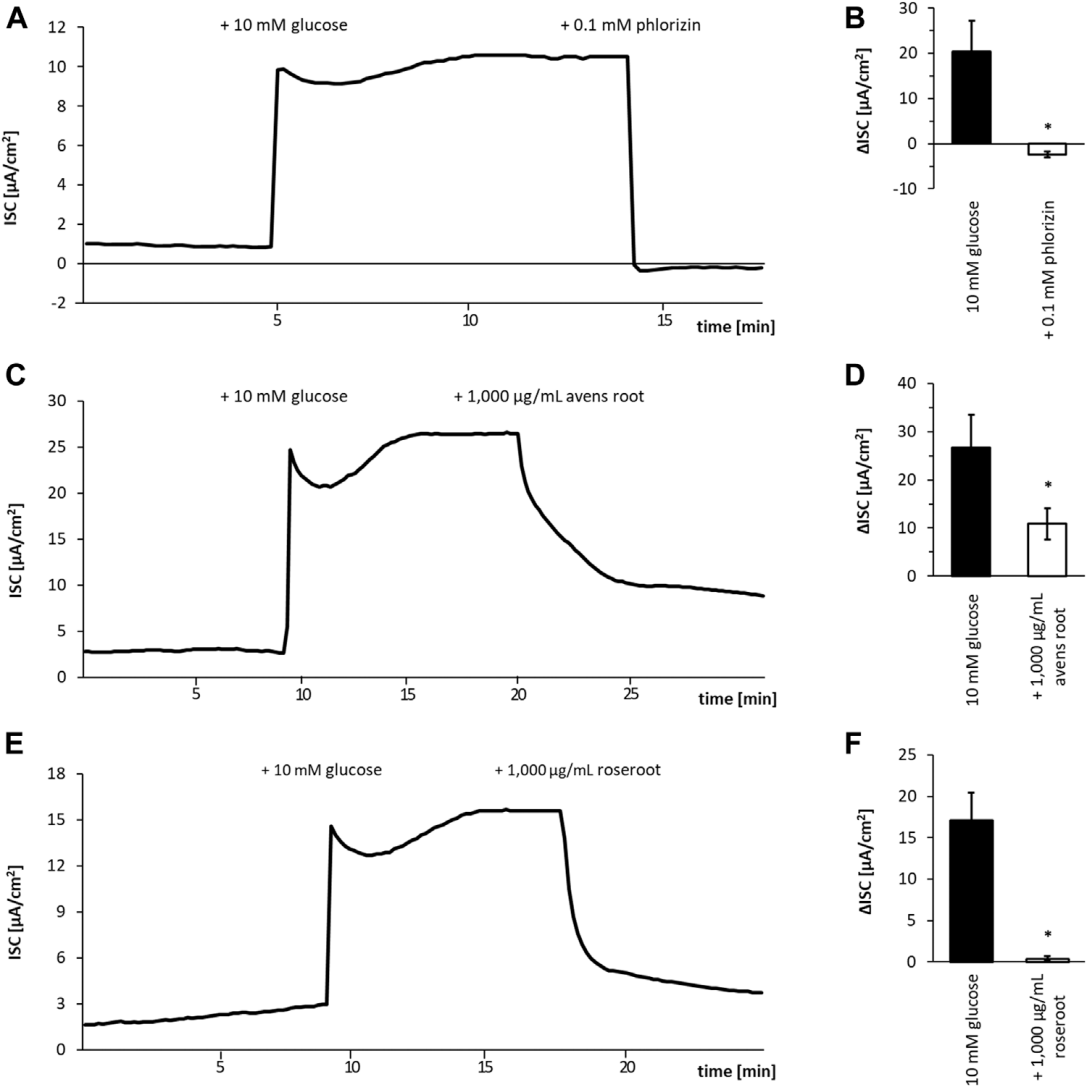
Influence of root extracts on SGLT1-dependent glucose transport in Caco-2 cell monolayers. Caco-2/PD7 monolayers were mounted in Ussing chambers and the short-circuit current (I_SC_) was monitored over time. Exemplary runs are depicted in **(A, C, E)**. The addition of glucose (10 mM) to the apical side led to a fast increase of the I_SC_. After the I_SC_ has reached a stable plateau (approximately 10 min after glucose addition), 1,000 μg/ml avens root extract **(A)** 1,000 μg/ml roseroot extract **(C)** or phlorizin (0.1 mM) **(E)** as positive control was added. The corresponding calculated I_SC_ values are shown in **(B, D, F)**. Error bars indicate the standard error of the mean. All experiments (n = 4).

To examine whether the selected root extracts affect the SGLT1-mediated glucose transport, the Caco-2/PD7 cell monolayer model was employed in Ussing chambers. Avens root extract at a concentration of 1,000 μg/ml led to an inhibition of the glucose-induced I_SC_ by 53.7%. Compared to this, roseroot extract (Figure 2) tested at the same concentration was found to be more effective in inhibiting SGLT1-dependent glucose transport with a 97.3% reduction of the glucose-induced I_SC_. As expected, 0.1 mM of the established SGLT1 inhibitor phlorizin (Ehrenkranz et al., 2005; Wright et al., 2011; Raja and Kinne, 2015) completely blocked the glucose induced I_SC_.

### Avens Root Extract, a Moderate Inhibitor of DPP4

**FIGURE 3:**
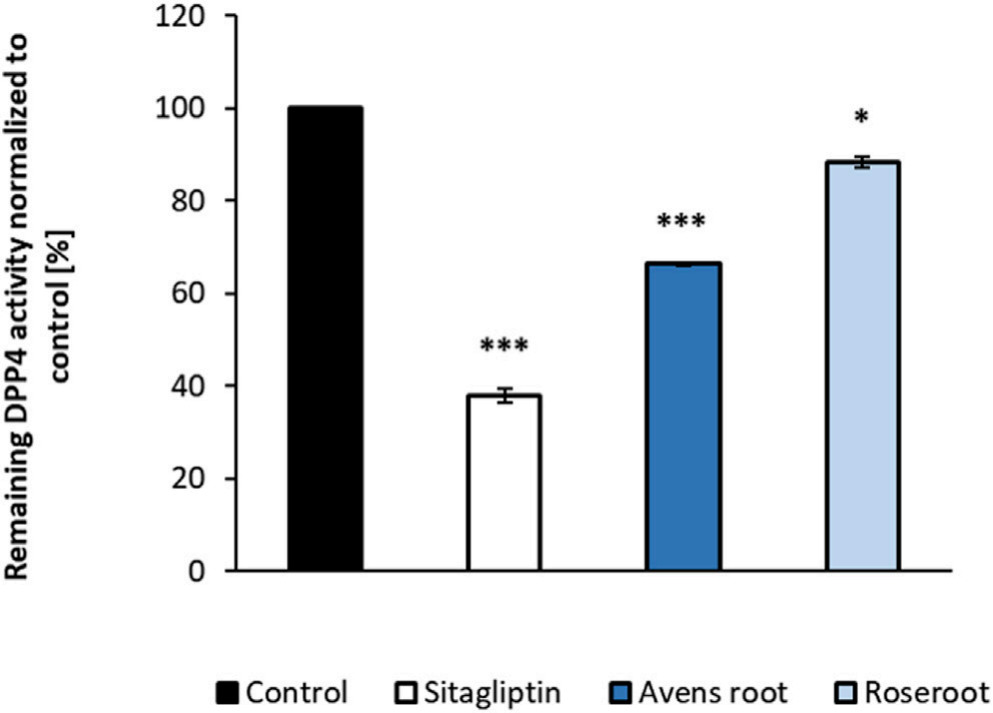
Influence of root extracts on the *in-vitro* activity of dipeptidyl peptidase-4 (DPP4) enzyme activity. DPP4 assays were carried out in the presence of the indicated substances (control: assay buffer; sitagliptin: 18 nM; root extracts: 1,000 μg/ml). The percentage values of remaining DPP4 enzyme activity in comparison to the control are shown. Results are mean values of n = 2 duplicate. Error bars indicate the standard deviation. ***p < 0.001, *p < 0.05, with post-hoc multiple comparison test of Dunnett.

Avens root extract tested at a final concentration of 1,000 μg/ml significantly decreased the DPP4 activity by 33.6% (*p* < 0.001) (Figure 3). In comparison, roseroot extract showed lower inhibitory activity (11.5%) at the same concentration. The positive control sitagliptin applied at its reported IC_50_ value of 18 nM reduced the DPP4 activity by 62.1%.

### Avens Root Extract, a Weak Stimulator of GLUT4 Translocation

**FIGURE 4:**
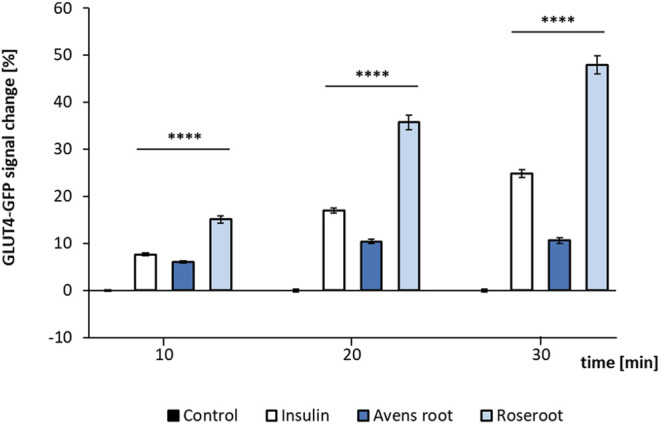
GLUT4-GFP translocation response to root extracts and insulin. GLUT4-myc-GFP cells were seeded in 96-well plates (40,000 cells per well), grown overnight followed by 3 h of starvation in Hank’s balanced salt solution (HBSS) buffer. Subsequently, the cells were stimulated by the addition of insulin (100 nM) or root extracts (1:10,000) dissolved in Krebs Ringer phosphate HEPES buffer (control) for 10–30 min. Results of two testing days are summarized. Control (n = 88); Insulin (n = 99); Avens root (n = 96); Roseroot (n = 72–83). Error bars indicate the standard error of the mean. ****p < 0.0001, with a significant change to control.

Both root extracts, when tested at a concentration of 1:10,000 led to a significant GLUT4 translocation (*p* < 0.0001) as determined by TIRF microscopy (Figure 4). Here, a time-dependent increase of the fluorescence signal intensity indicated GLUT4 translocation. Avens root extract led to a moderate signal increase by 10.6% after 30 min incubation. Remarkably, the addition of roseroot extract resulted in a signal considerably higher than the positive control insulin (48.0% compared to 24.8% after 30 min). Moreover, a clear time-dependent increase of the signal, comparable to the effect of insulin, was observed. In order to eliminate false positive hits caused by auto-fluorescence (Stadlbauer et al., 2020), the GFP signal change after incubation with the extracts in cell-free regions was evaluated (data not shown).

### Avens Root Extract Lowered Blood Glucose Levels *In-Ovo*

**FIGURE 5:**
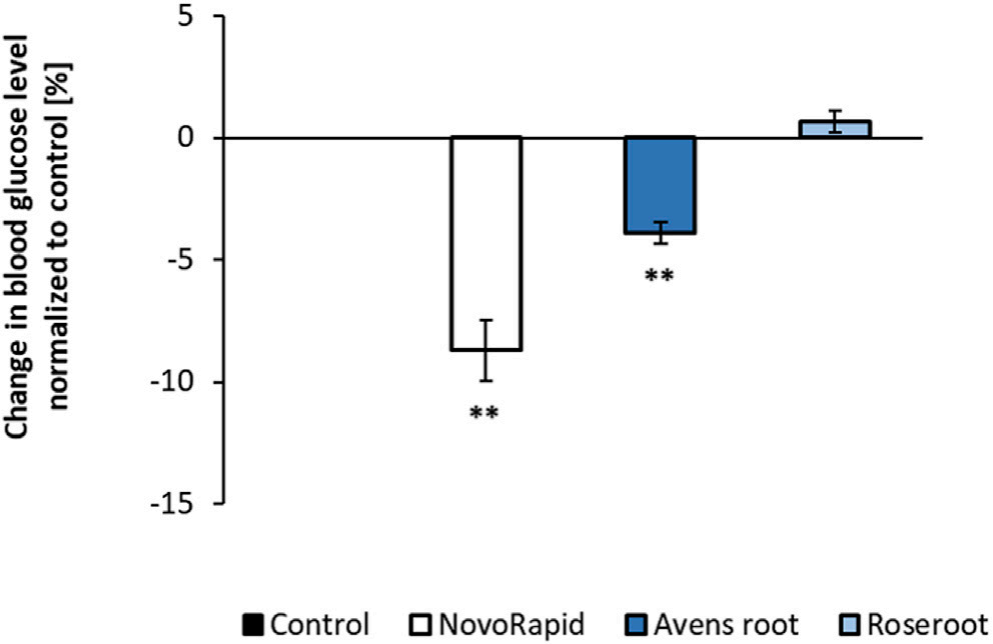
Influence of root extracts on blood glucose levels *in-ovo*. The air compartment of 11 day old hen’s eggs were treated with ddH_2_O (control) or the indicated substances (NovoRapid: 3 U/ml; root extracts: 1:17) dissolved in ddH_2_O (300 µl volume) for up to 60 min. After that, a suitable blood vessel of the chicken embryo was dissected for blood collection. Blood glucose levels were determined with a blood glucose meter. Control (n = 4); NovoRapid (n = 5); avens root extract (n = 4); roseroot extract (n = 4). Error bars indicate the standard error of the mean. **p < 0.01, with a significant decrease with respect to control.

To test the impact of the selected extracts on the blood glucose level in a living organism, the recently established HET-CAM model was used (Haselgrübler et al., 2017; 2018b). As shown in Figure 5, application of 600 μg/ml avens root extract significantly lowered the glucose status by 3.9% after 60 min (*p* < 0.01). However, no significant effect on blood glucose levels occurred when 600 μg/ml roseroot extract was added. Compared to this, 3°U/ml of the positive control, the insulin analogue NovoRapid resulted in a significant reduction of blood glucose level by 8.7% after 60 min incubation time (*p* < 0.01).

### Avens Root Extract Supplementation Reduced Dietary Starch Induced Triglyceride Accumulation in *D. melanogaster*

**FIGURE 6:**
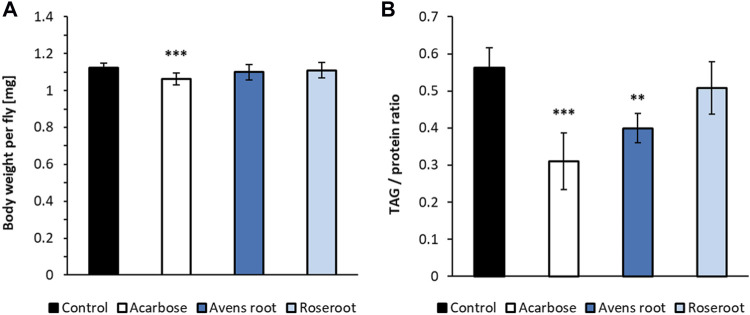
The impact of root extracts on body weight and lipid storage in *D. melanogaster*. Female *D. melanogaster* were raised on a 10% starch, 4% yeast extract diet supplemented with 1.8 μg/ml acarbose, 2.5% avens root and 2.5% roseroot extract, respectively. The body weights **(A)** and triglyceride to protein ratios **(B)** were determined on day 10 after eclosion and compared to the respective values for flies fed a control diet without supplement. Bars represent the mean ± standard deviation of three independent experiments performed in triplicate. Statistical analysis was carried out by using one-way ANOVA followed by Dunnett’s multiple comparisons test (**p < 0.01; ***p < 0.001).

Supplementation of a starch-based *Drosophila* diet with 2.5% avens root extract led to a significantly reduced triglyceride content in 10°days old female flies when compared to control animals. The body weight was not affected (Figures 6A,B). Treatment with the positive control acarbose resulted in a similar decline in lipid storage. However, for these animals a slightly reduced body weight was determined. In contrast to that and in line with a previous report (Schriner et al., 2016), 2.5% roseroot extract did not alter the triglyceride level and body weight of female flies.

### Identification of Phenolic Compounds in Root Extracts

We next determined the TPC of the selected root extracts, since phenolic compounds are prime candidate molecules with respect to the observed bioactivities. The TPC of the avens root extract was determined to be 7,249 ± 132 mg GAE/L and, hence, slightly higher than the content in the roseroot extract with 6,080 ± 166 mg GAE/L (means ± SEM of three independent determinations).

**FIGURE 7:**
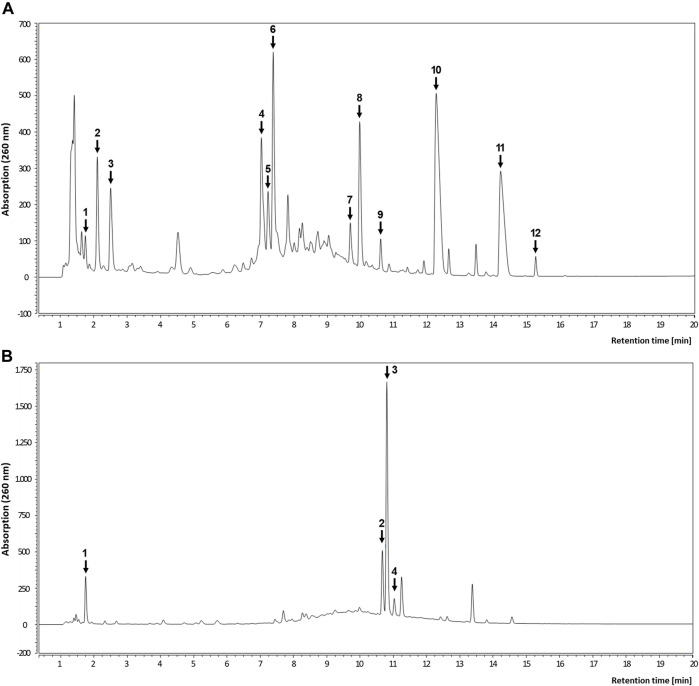
Representative HPLC analyses of root extracts. Chromatogram of avens root extract **(A)** and roseroot extract **(B)** recorded at 260 nm. For peak identification, refer to Table 3.

Subsequently, the root extracts were further analyzed for specific phenolic constituents by using HPLC with DAD detection and HPLC-MS, respectively (Table 3; Figure 7). The polyphenolic composition of the avens root extract was characterized by a high content of ellagic acid and its derivatives as well as ellagitannins such as pedunculagin, stenophyllanin, stachyurin, casuarinin and gemin A. In line with literature data on *R. rosea* (Granica et al., 2016; Olennikov et al., 2020; Pawłowska et al., 2020), rosarin was confirmed as a main constituent of roseroot extract.

**TABLE 3:** Identification of major compounds in root extracts using LC-MS analysis.

Sample	Peak number	Retention time t_R_ [min]	Compound	Mass spectrometry (M-H)^-^ [*m/z*]
Avens root	1	1.74	Gallic acid	169.0215
	2	2.10	Pedunculagin 1	783.1021
	3	2.50	Pedunculagin 2	783.1026
	4	7.02	Stenophyllanin A	1207.1970
	5	7.22	Stachyurin	935.1176
	6	7.37	Casuarinin	935.1196
	7	9.60	Gemin A	935.1213
	8	9.97	Ellagic acid	300.9992
	9	10.59	Dimethyl-O-ellagic acid	329.0313
	10	12.26	Dimethyl-O-ellagic acid	329.0308
	11	13.45	Dimethyl-O-ellagic acid	329.0310
	12	14.19	Trimethyl-O-ellagic acid	343.0465
Roseroot	1	1.75	Gallic acid	169.0214
	2	10.60	Rosarin isomer	—
	3	10.80	Rosarin	427.1794
	4	11.10	Rosarin isomer	—

Identification based on high-resolution MS data and comparison to literature.

## Discussion

In the present study, we have employed a collection of *in-vitro* assays representing various crucial steps within the glucose metabolism from intestinal digestion to glucose uptake in peripheral tissues and two alternative, not widely used *in-vivo* models for the identification and evaluation of candidate plant root extracts with antidiabetic activity.

Starting with a target-based screening for α-glucosidase inhibitors, we identified two promising aqueous root extracts, namely *G. urbanum* (avens root) and *R. rosea* (roseroot) out of a sub-library of 111 root extracts derived from a local plant extract collection PECKISH (Onur et al., 2013). As indicated by the prespective IC_50_ values, the potency of avens root and roseroot extracts to inhibit α-glucosidase activity exceeded that of the established antidiabetic drug acarbose, an oligosaccharide of microbial origin (Actinoplanes), by a factor of approximately 100. Both, *R. rosea* and *G. urbanum* are traditional medicinal plants. However, in contrast to roseroot that has been already frequently reported to exert antidiabetic activity (Apostolidis et al., 2006; Kim et al., 2006; Kwon et al., 2006; Christensen et al., 2009; Niu et al., 2014; Bai et al., 2019; Pu et al., 2020), a PubMed search for the term “*Geum urbanum* and diabetes” revealed solely one publication, where *Geum urbanum* L. was part of a polyherbal mixture that exhibited anti-hyperglycemic activity in a rat model (Madić et al., 2021). Being excellent inhibitors of α-glucosidase activity, we also tested whether a combination of avens root and roseroot extract show synergistic inhibitory effects. This is of interest, since lower doses of individual extracts used in combination may reduce potential side effects (Tallarida, 2011). Our analyses revealed an additive effect of the two extracts with respect to α-glucosidase inhibition.

Similar to acarbose and in line with previous reports (Apostolidis et al., 2006; Kwon et al., 2006), roseroot extract was also a potent inhibitor of α-amylase, whereas avens root extract turned out to be inefficient here. Several secondary plant metabolites have been demonstrated to bind unspecifically to proteins and other biomolecules thereby affecting numerous targets. Such molecules are classified as PAIN compounds (pan-assay interference compound) (Baell and Holloway, 2010) and are known for often giving false positive results in high-throughput screens. In this regard, the specific action of the *G. urbanum* root extract on α-glucosidase without affecting α-amylase activity is remarkable.

Targeting multiple points within the glucose metabolism may increase the efficiency of plant extracts in terms of lowering the postprandial blood glucose level. Hence, we tested the impact of the two identified root extracts on other promising antidiabetic targets, namely SGLT1 (Song et al., 2016), DPP4 (Papaetis, 2014) and GLUT4 (Govers, 2014). We found that, again, roseroot extract reduced the SGLT1-mediated glucose transport more efficiently than avens root extract. SGLT1 activity that is responsible for the absorption of dietary glucose from intestinal lumen into enterocytes has been mostly determined by using isolated intestinal tissue from sacrificed animals in Ussing chambers (Clarke, 2009). Alternatively, monolayers of the human colorectal adenocarcinoma Caco-2 cell line and labeled glucose/glucose analogues have been employed to measure SGLT1-mediated transport (Zheng et al., 2012; Steffansen et al., 2017). However, combining Ussing chamber technique with Caco-2 cell culture to study SGLT1 mediated glucose uptake has been rarely reported (Yin et al., 2014). In accordance with this previous report, we confirm that Caco-2 monolayers mounted in Ussing chambers represent an excellent model to study pharmacological extracts/compounds that target the SGLT1 mediated glucose transport. Of note, different Caco-2 cell clones exist and they are characterized by drastic differences in SGLT1 expression. Accordingly, we chose the Caco-2 clone PD7 which has been reported to exhibit the highest SGLT1 expression level of all examined Caco-2 clones (Mahraoui et al., 1994).

DPP4 inhibition prevents the degradation of the incretin hormones glucagon-like peptide 1 (GLP-1) and gastric inhibitory polypeptide (GIP), thereby lowering blood glucose levels (Papaetis, 2014). Although roseroot as well as avens root significantly reduced DPP4 activity when tested at a final extract concentration of 1,000 μg/mL, it remains questionable whether such concentrations can be achieved *in-vivo*.

When tested in a GLUT4-GFP reporter cell line, we found that comparatively low concentration (Haselgrübler et al., 2018a) of avens root or roseroot extract (1:10,000) led to a significant response, which in case of the roseroot extract was comparable to the effect of the positive control insulin. GLUT4 translocation into the cell membrane is a key event for the uptake of blood glucose into adipose tissues and striated muscles. The roseroot data are in good accordance with previous reports, where intraperitoneal injection of an aqueous *R.rosea* extract led to an increased GLUT4 expression in skeletal muscle of diabetic rats (Niu et al., 2014). In accordance with that, salidroside, a main constituent of *R. rosea* (Panossian et al., 2010; Li et al., 2017; Dimpfel et al., 2018), promoted the glucose uptake in adipocytes (Wang et al., 2004).

When summarizing our *in-vitro* data, the roseroot extract hit more of the tested targets than the avens root extract and in most assays was even more potent in terms of its inhibitory activity. However, when the root extracts were evaluated in two *in-vivo* models, we found that solely the avens root extract exhibited antidiabetic activity. In the first model, a modified hen’s egg test (Gluc-HET), application of the avens root extract led to significantly reduced blood glucose levels, which was comparable to the effect of the positive control (the insulin analogue NovoRapid), whereas no change in blood glucose was observed for the roseroot extract. In contrast to this, Niu et al., 2014 showed that an aqueous *R. rosea* extract dose-dependently lowered the plasma glucose level in streptozotocin-induced diabetic rats. Of note, in this study the extract was applied by intraperitoneal injection, which may result in a higher bioactivity. However, we cannot rule out that the reported blood glucose lowering effect of roseroot extract may be specific for mammals.

In the second model, *D. melanogaster*, supplementation of 2.5% avens root extract but not of 2.5% roseroot extract resulted in the reduction of the triacylglyceride level in female fruit flies that were fed a starch-rich diet. In good accordance with this, Schriner et al. (2016) reported that when supplementing the *Drosophila* diet with the same concentration of roseroot extract, the fat and protein levels of fruit flies were not affected independent on the carbohydrate content of the diet (0.09% vs. 9% sucrose). However, the roseroot extract elevated the sugar content of the flies on the low-carbohydrate diet.

Our determination of the TPC in combination with HPLC analyses revealed that phenolic compounds with potential biological activity are abundant in the two selected root extracts. For the aqueous *R. rosea* extract, rosarin and rosarin isomers were identified as main constituents, which is in line with previous analytical studies. However, other phenolic compounds that have been frequently determined in roseroot extracts, including salidroside, a phenyl-ethanoid and rosavins (phenyl-propanoids) (Panossian et al., 2010; Li et al., 2017; Dimpfel et al., 2018; Olennikov et al., 2020) did not show up in our analyses. Of note, Dimpfel et al. (2018) analysed different roseroot preparations and found variations in the content of active ingredients including salidroside and rosavins. Factors that are known to affect herbal preparations are the geographic origin, the harvesting season, the drying procedure and the extraction method. Among these compounds, particularly salidroside has been shown to exhibit antidiabetic properties both, in *in-vitro* and *in-vivo* studies. For example, when tested in the L6 myoblast cell line, salidroside treatment was found to elicit an enhanced glucose uptake (Li et al., 2008). Furthermore, Zheng et al., 2015 reported antidiabetic effects of orally applied salidroside on obese *db/db* mice including decreased blood glucose levels, an improved glucose tolerance, increased insulin sensitivity and enhanced GLUT4 expression in skeletal muscles. With regard to the underlying molecular mechanism, in-vitro experiments using hepatocyte and myoblast cell lines indicated that the AMPK related signalling pathway is a target of salidroside (Li et al., 2008; Zheng et al., 2015). Accordingly, the absence of detectable salidroside concentrations in our roseroot extract is a possible explanation for the lack of an antidiabetic activity in the corresponding *in-vivo* models.

Regarding major phenolic compounds of the aqueous avens root extract, our analyses revealed ellagic acid and its derivatives as well as ellagitannins such as pedunculagin, stenophyllanin, stachyurin, casuarinin and gemin A. This is in line with previous studies on *G. urbanum* extracts (Owczarek et al., 2015; Granica et al., 2016; Neshati et al., 2018; Al-Snafi, 2019). In particular, ellagic acid and its derivatives have been suggested to be very promising agents against DM and diabetic complications as recently summarized (Amor et al., 2020). Some effects reported herein are of great interest in the context of our study: First, a α-glucosidase inhibition by ellagic acid-rich plant extracts is described which is far more effective than the inhibition of α-amylase (Kam et al., 2013; Yin et al., 2014; Bellesia et al., 2015). Second, one study reported DPP4 as a putative target of ellagic acid (Mohanty et al., 2019). And third, a significantly reduced blood glucose level by an ellagic acid-rich plant extract was observed in a diabetic mouse model which was tested in an oral glucose tolerance test (Abu-Gharbieh and Shehab, 2017). Remarkably, all these effects are in line with the outcome of our study in terms of avens root extract activity. Besides, Granica et al., 2016 postulated that gemin A is the active compound of *G. urbanum*. In this study, gemin A, detected as main constituent, showed high anti-inflammatory potential. However, there are no data on the underlying mechanisms or on gemin A in the context of diabetes.

The discrepancy between our *in-vitro* and *in-vivo* results on roseroot and avens root bioactivity have been frequently observed in comparable studies (Schrader et al., 2012). This underlines the need to further evaluate the efficacy of candidate extracts that have been identified by enzyme- or cell culture-based assays in studies with living organisms in order to address the pharmacology of the relevant bioactive compounds. We suggest that non-rodent models such as hen’s eggs or the fruit fly *D. melanogaster* can be integrated into this process, since they allow a cheap and rapid *in-vivo* evaluation without major ethical considerations. The HET CAM model represents a powerful system for the analysis of potential insulin mimetic compounds. Chicken embryos used on day 11 of the development are insulin sensitive, but do not produce insulin at this stage. Importantly, chicken embryos are vertebrates and therefore evolutionary more close to humans than invertebrate models. However, at day 11 they do not possess a fully developed nervous system and thus no pain perception. It is therefore regarded a valuable alternative model reducing the number of animals in well established rodent models such as mice and rats (Haselgrübler et al., 2017). *D. melanogaster* represents a valuable complementation to the HET CAM model, since it includes pharmacological aspects such as bioavailability, the influence of the gut microbiota and biotransformation that cannot be covered by the hen’s egg test. Moreover, we suggest that when obtaining consistent antidiabetic activities in such different models (an invertebrate and a vertebrate), it is more likely that these findings can be transferred to other organisms including humans. In this regard, we would like to add that although the general steps of glucose metabolism are evolutionary highly conserved, we are aware that our alternative *in-vivo* models hen’s egg and the fruit fly have their limitations. Here, we have demonstrated that the combination of an *in-vitro* tool box of assays and two *in-vivo* models is suitable to unveil novel potent plant extracts derived from a plant extract library. Nevertheless, it is obvious that the antidiabetic potential of avens root extract needs to be further verified in rodent models or human intervention studies, which still represent the gold standard for this purpose.

## Data Availability

The raw data supporting the conclusions of this article will be made available by the authors, without undue reservation.

## Abbreviations

*D. melanogaster*, *Drosophila melanogaster*; DM, diabetes mellitus; DPP4, dipeptidyl peptidase-4; *G. urbanum*, *Geum urbanum* L.; GLUT4, glucose transporter 4; HET-CAM, hen`s egg test-chorioallantoic membrane; HPLC-MS, high-performance liquid chromatography-mass spectrometry; PECKISH, plant extract collection Kiel in Schleswig-Holstein; *R.rosea*, *Rhodiola rosea* L.; SGLT1, sodium-dependent glucose transporter 1; TEER, transepithelial electrical resistance; TIRF, total internal reflection fluorescence; TPC, total phenolic content.
