# Supplementary material for: Avens Root (Geum Urbanum L.) Extract Discovered by Target-Based Screening Exhibits Antidiabetic Activity in the Hen’s Egg Test Model and Drosophila melanogaster
Source: Front Pharmacol. 2021 Dec 15;12:794404. doi: 10.3389/fphar.2021.794404 (PMC8715001; doi:10.3389/fphar.2021.794404)
Supplement: Supplementary file 1 [file DataSheet1.docx]

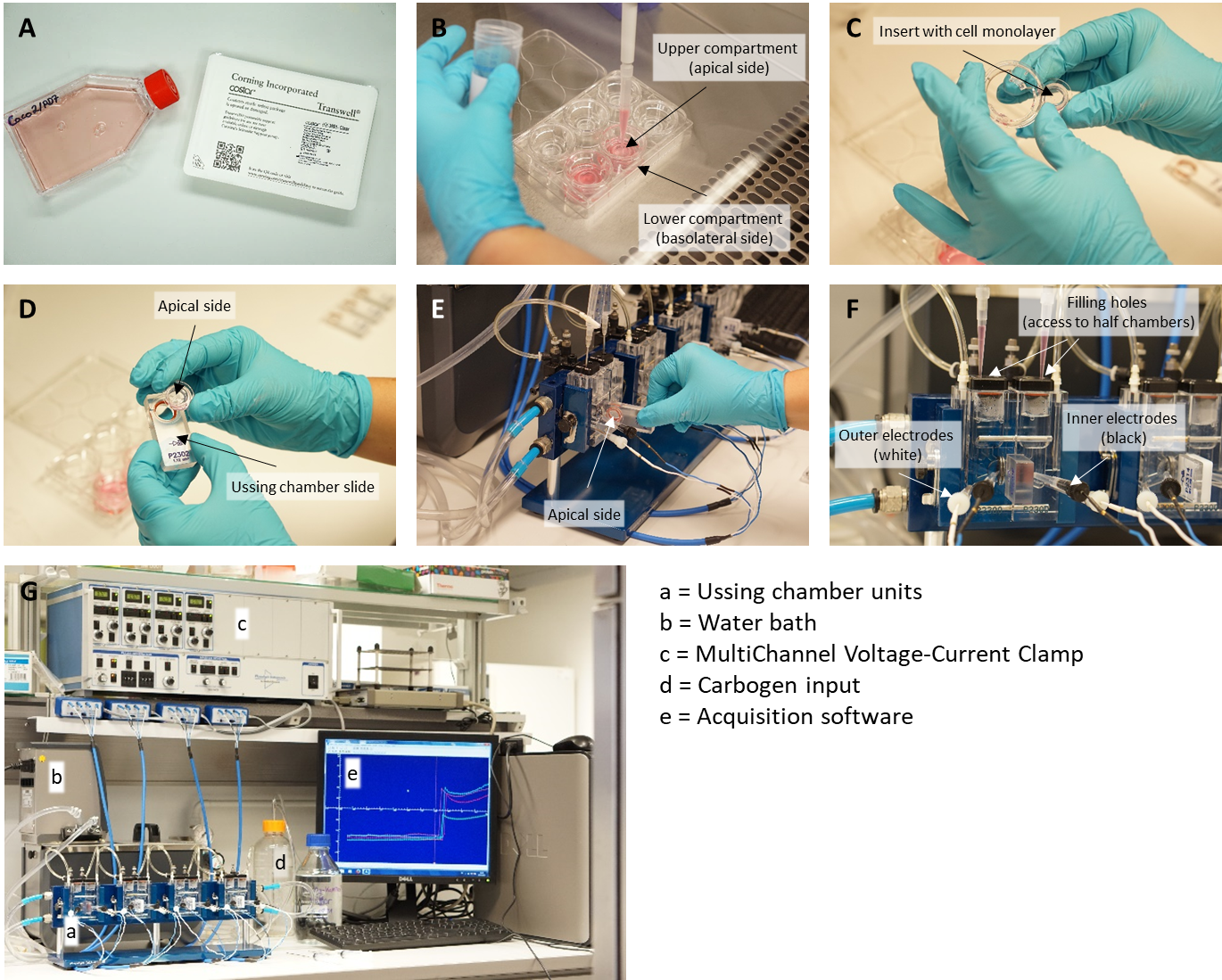


**Figure S1: Experimental procedure of Ussing chamber experiments. (A)** The Caco-2/PD7 clone and 6-well Corning® Costar® Snapwell cell culture plates were used for transport studies. **(B)** Cells were seeded into the upper compartment of 6-well Snapwell cell culture inserts. Cell-free medium was given into the lower compartment. **(C-E)** After 21 days of cultivation, the Snapwell inserts with the Caco-2/PD7 monolayers were transferred to Ussing chamber slides and mounted in the Ussing chamber. **(F)** Both half-chambers of a Ussing chamber unit were filled with 5 ml of Henk’s balanced salt solution (HBSS) containing 10 mmol/L mannitol apically and 10 mmol/L glucose basolaterally maintained at 37 °C and continuously carbogen bubbled. To enable the addition of further solutions, the half chamber are accessible via the indicated filling holes. The transepithelial potential difference was continuously monitored under circuit conditions using a DVC 1000 amplifier (WPI) and recorded through Ag-AgCl electrodes (inner black electrodes) and HBSS agarose bridges. The short-circuit current (I_SC_) was measured via an automatic voltage clamp (outer white electrodes). **(G)** The set-up of the Ussing chamber workplace is shown. Recordings were collected and stored using the A&A II acquisition software. To test the influence of root extracts on SGLT1-dependent glucose transport in Caco-2 monolayers glucose were added apically to stimulate Na^+^-coupled glucose transport. When the glucose-stimulated I_SC_ reached a stable plateau, root extracts or phlorizin as positive control were added. I_SC_ values were further recorded until they reached a stable level. The average I_SC_ of 2 min intervals within stable plateaus were used to calculate differences in SGLT1 transport activity.


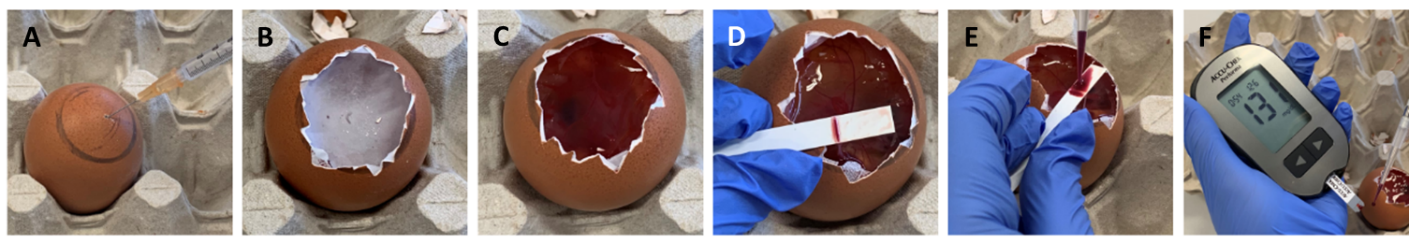


**Figure S2: Experimental procedure of the hen’s egg test. (A)** The air bladder was marked on the eggshell, and **(B)** the chorioallantoic membrane of chicken embryos was incubated with the root extracts for 60 min. **(C)** The eggshell and the egg membrane were removed, **(D)** the chorioallantoic membrane was cut, and **(E)** blood was collected from a main vessel. **(F)** A blood glucose meter was used for measurements (reproduced from Stadlbauer et al., 2021).
